# Supplementary material for: Compressed sensing-based approach identifies modular neural circuitry driving learned pathogen avoidance
Source: eLife. 2026 Feb 10;13:RP97340. doi: 10.7554/eLife.97340 (PMC12890250; doi:10.7554/eLife.97340)
Supplement: Supplementary file 1. [file elife-97340-supp1.docx]

**Supplementary Table 1.** Archaerhodopsin lines that constitute the measurement matrix

| **Promoters** | **Neurons** |
| --- | --- |
| wSR853: [pdop-2::Arch-tagRFP]; sraIs[pstr-2::mkO]; lite-1(ce314)x | CEP; SIA; SIB; RIA; RID; ADE |
| wSR517: [pflp-21::Arch-tagRFP, pBX]; pha-1(e2123)III; lite-1(ce314)x | URX; URA; RMG; MC; M2; AIY |
| sraEx280[pttx-3::Arch-tagRFP; pBX]; pha-1(e2123)III; lite-1(ce314)x | AIY |
| wSR230: [pstr-2::Arch-tagRFP; pBX]; pha-1(e2123)III; lite-1(ce314)x | AWC |
| wSR347: [pflp-3::Arch-GFP; pflp-3::mKO; pBX]; pha-1(e2123)III; lite-1(ce314)x | IL1; OLL; URB; PQR |
| wSR469: [pflp-19::Arch-tagRFP]; pha-1(e2123)III; lite-1(ce314)x | AWA; URX |
| wSR495: [pmpz-1prom2::Arch-tagRFP]; pha-1(e2123)III; lite-1(ce314)x | M4; NSM; MC; RMH; RMF; RMD; HSN; SDQ; PVC; PVQ; PVN |
| wSR545: [pflp-11::Arch-tagRFP, pBX]; pha-1(e2123)III; lite-1(ce314)x | AUA; BAG; DVB; LUA; PHC; PVC; SAB; URX |
| wSR523: [pflp-12::Arch-tagRFP, pBX]; pha-1(e2123)III; lite-1(ce314)x | BAG; SAA; SMB; AVH; AVJ |
| wSR496: [pflp-4::Arch-tagRFP]; pha-1(e2123)III; lite-1(ce314)x | NSM; ADL; I5; I6; AWC; FLP |
| wSR512: [pinx-4:Arch-tagRFP, pBX]; pha-1(e2123)III; lite-1(ce314)x | ADA; ADE; AIN; AUA; AVJ; DVC; FLP; PHA; PHB; PVR; PVT; RIC; RIG; RIM; RIP |
| wSR500: [pflp-7::Arch-tagRFP]; pha-1(e2123)III; lite-1(ce314)x | ALA; AVG; PHB; PDA; PVW; RIC; SAA |
| wSR683: [pmod-1::Arch-tagRFP, pBX]; sraIs467[pstr-2::mKO] III; lite-1(ce314)x; pha-1(e2123) III | RID; RME; AIZ; AIY; DD1 |
| wSR448: [pmbr-1::Arch-tagRFP]; pha-1(e2123)III; lite-1(ce314)x | AWC; AIM; RIC; AIN |
| wSR274: [pser-2prom2::Arch-tagRFP; pBX]; pha-1(e2123)III; lite-1(ce314)x | RME; AIZ; RID; AIY; BDU |
| wSR454: [plin-11::Arch-tagRFP]; pha-1(e2123)III; lite-1(ce314)x | ADF; ADL; AIZ; RIC; AVG; AVH; AVJ |
| wSR186: [psra-11::Arch-GFP, psra-11::mKO]; lite-1(ce304) x | AIY; AVB; AIA |
| wSR453: [pmgl-1::Arch-tagRFP]; pha-1(e2123)III; lite-1(ce314)x | AIA; RMD; NSM |
| wSR345: [popt-3::Arch-GFP; popt-3::mKO; pBX]; pha-1(e2123)III; lite-1(ce314)x | DVA; AVE; ASJ; OLQ; AIM; CAN |
| wSR507: [pnmr-1:Arch-tagRFP]; pha-1(e2123)III; lite-1(ce314)x | AVA; AVD; AVE; RIM; AVG |
| wSR241: [prig-5::Arch-tagRFP; pBX]; pha-1(e2123)III; lite-1(ce314)x | RMD; SMD; I2; MC; M4 |
| wSR352: [pnpr-4::Arch-GFP; pnpr-4::mKO; pBX]; pha-1(e2123)III; lite-1(ce314)x | SIA; SIB; RIC; AVA; RMD; AIY; AVK; BAG |
| wSR550: [pser-2prom3::Arch-tagRFP, pBX]; pha-1(e2123)III; lite-1(ce314)x | OLL; PVD |
| wSR224: [podr-2(16)::Arch-tagRFP; pBX]; pha-1(e2123)III; lite-1(ce314)x | SMD; RME |
| wSR486: [psams-5::Arch-tagRFP];pha-1(e2123)III; lite-1(ce314)x | MI; PVQ |
| wSR468: [pgpa-14::Arch-tagRFP; pbx]; pha-1(e2123)III; lite-1(ce314)x | ASI; ASJ; ASH; ASK; ADE; PHA; PHB; ALA; AVA; CAN; DVA; PVQ; RIA |
| wSR288: [podr-2(18)::Arch-tagRFP; pBX]; pha-1(e2123)III; lite-1(ce314)x | SMB; RME; ALN; PLN; RIG |
| wSR535: [pmgl-3:Arch-tagRFP, pBX]; pha-1(e2123)III; lite-1(ce314)x | NSM; ADF; ASE; AWC; RIB; RIC; BAG |
| wSR499: [pflp-22::Arch-tagRFP]; pha-1(e2123)III; lite-1(ce314)x; line-3 | AIM; ASG; AVA; AVG; AVL; CEP; PVD; PVW; RIC; AIZ; RIV; SMD; URA |
