# Supplementary material for: Compressed sensing-based approach identifies modular neural circuitry driving learned pathogen avoidance
Source: eLife. 2026 Feb 10;13:RP97340. doi: 10.7554/eLife.97340 (PMC12890250; doi:10.7554/eLife.97340)
Supplement: Supplementary file 2. [file elife-97340-supp2.docx]

**Supplementary Table 2.** Channelrhodopsin and Halo lines

| **Promoters** | **Neurons** |
| --- | --- |
| wSR281: [pttx-3::ChR2-tagRFP; pBX]; pha-1(e2123); lite-1(ce314)x | AIY |
| wSR491:[pnpr-4::ChR2-tagRFP]; pha-1(e2123)III; lite-1(ce314)x | SIA; SIB; RIC; AVA; RMD; AIY; AVK; BAG |
| ZX888: zxIs16 [pflp-1::NpHR::eCFP; lin-15+] | AVK |
