# Supplementary material for: Compressed sensing-based approach identifies modular neural circuitry driving learned pathogen avoidance
Source: eLife. 2026 Feb 10;13:RP97340. doi: 10.7554/eLife.97340 (PMC12890250; doi:10.7554/eLife.97340)
Supplement: Supplementary file 3. [file elife-97340-supp3.docx]

**Supplementary Table 3.** GCaMP Lines

| **Promoters** | **Neurons Imaged** |
| --- | --- |
| wSR762: pnpr-4::GCaMP6s; pstr-2::mKO; lite-1(ce314)x | AVK,SIA |
| wSR490: sraEx490[pttx-3::GCaMP6s(mammalian)]; sraIs467[pstr-2::mKO]; lite-1(ce314) | AIY |
